# Fermented Tea and Cognitive Dysfunction in Diabetes: A Novel Perspective on the Gut‐Brain AXIS

**DOI:** 10.1002/fsn3.71048

**Published:** 2025-10-12

**Authors:** Ruyi Zhang, Wenli Liao

**Affiliations:** ^1^ Basic Medical School Hubei University of Science and Technology Xianning P.R. China; ^2^ School of Pharmacy Hubei University of Chinese Medicine Wuhan P.R. China

**Keywords:** *Chibi Green Brick tea*, diabetic cognitive dysfunction, fermented tea, gut microbiota, gut‐brain axis, microbial metabolites, polyphenols

## Abstract

The interplay among diabetes, cognitive decline, and the gut microbiome represents an emerging field of scientific inquiry. The gut‐brain axis serves as a crucial communication network between the gastrointestinal system and the central nervous system, playing a significant role in diabetes‐related cognitive deterioration. Fermented tea, enriched with bioactive constituents, offers a promising therapeutic strategy by modulating this axis. Specifically, compounds including catechins in fermented tea positively modulate gut microbiota composition, promoting commensal bacteria while suppressing pathogenic strains. This microbial shift enhances the production of short‐chain fatty acids, which may strengthen gut barrier integrity, attenuate systemic inflammation, and thereby influence cognitive health via the gut‐brain axis. Concurrently, the antioxidant properties of tea polyphenols and catechins mitigate oxidative stress, a key pathogenic factor in diabetic cognitive impairment. Furthermore, the anti‐inflammatory effects of fermented tea may potentially ameliorate chronic low‐grade inflammation in diabetes, offering a plausible pathway for cognitive improvement. This systematic review, conducted through comprehensive searches of PubMed, Web of Science, CNKI (China National Knowledge Infrastructure), and Wanfang databases, synthesizes evidence supporting fermented tea as a natural intervention to preserve cognitive function in diabetic individuals by targeting the gut microbiome and the gut‐brain axis. Notably, it also explores the potential application of *Chibi Green Brick tea* as a regionally specific intervention for diabetes‐related cognitive dysfunction. Collectively, these insights underscore the necessity for rigorous mechanistic investigations and robust clinical validation to fully elucidate therapeutic mechanisms of fermented tea and translate these findings into clinical practice.

## Graphical abstract

Fermented tea can treat cognitive dysfunction in diabetes through the gut‐brain axis. The green brick tea from Chibi, Xianning City, Hubei Province, China, has unique therapeutic potential.

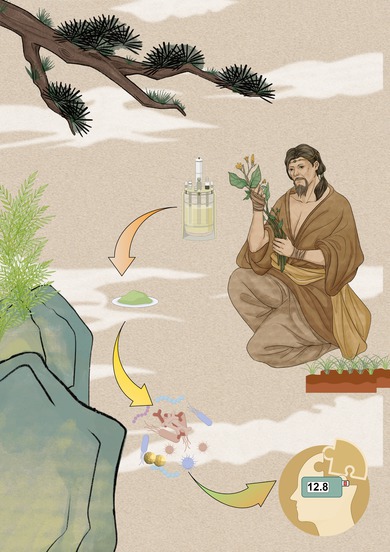

## 1 Introduction

Diabetes mellitus (DM) is a chronic metabolic disorder characterized by persistent hyperglycemia, posing a significant global health burden with escalating prevalence (Zhang et al. 2023; Biessels and Despa 2018). This condition impairs insulin production and signaling, leading to multisystem complications including cognitive dysfunction (Song et al. 2022). DM‐associated cognitive impairment represents a complex pathological condition, involving multiple mechanisms including oxidative stress, neuroinflammation, and disruption of the gut‐brain axis (Góralczyk‐Bińkowska et al. 2022; Xu et al. 2022; Borse et al. 2021; Loh et al. 2024; Pelle et al. 2023). The gut‐brain axis, a bidirectional signaling pathway integrating gastrointestinal tract and central nervous system functions, is crucial in the pathogenesis of cognitive decline in diabetic individuals (Longo et al. 2023; Socała et al. 2021). Emerging evidence underscores the critical influence of gut microbiota on neurological outcomes, suggesting microbiota‐targeted interventions may mitigate diabetes‐related cognitive deficits.

Tea, one of the most consumed beverages worldwide, exhibits diverse bioactive properties including antioxidant, anti‐inflammatory, and neuroprotective effects (Khan and Mukhtar 2018; Xu et al. 2021; Omagari et al. 2018; Jakubczyk et al. 2023). Fermented teas, in particular, have garnered significant research interest due to their unique profiles of polyphenols and other bioactive constituents, which may modulate cognitive functions through gut microbiome interactions (Khan and Mukhtar 2018; Luo et al. 2023; Li et al. 2019; Hong et al. 2022). Classification based on oxidation levels yields distinct categories (light, semi‐, full‐, and post‐fermented), each demonstrating characteristic phytochemical compositions and physiological activities (Hayat et al. 2015; Li et al. 2024). Notably, post‐fermented Pu‐erh tea demonstrates gut microbiota‐modulating properties and metabolic benefits (Li et al. 2017; Jeong et al. 2020; Zhang, Cheng, and Zhang 2021).

This review aims to critically examine the potential of fermented tea, specifically its bioactive tea polyphenols, in mitigating diabetes‐associated cognitive dysfunction by modulating the gut‐brain axis. We critically evaluate mechanistic pathways, including their influence on gut microbiota composition, metabolite production (e.g., short‐chain fatty acids, SCFAs), as well as attenuation of neuroinflammation and oxidative stress. Furthermore, we explore the therapeutic promise of *Chibi Green Brick tea*‐a traditional fermented tea from Hubei Province, China—noting its distinct microbial profile and fermentation process. Its documented antioxidant capacity and microbiota‐regulating effects suggest novel pathways for mitigating neurocognitive deficits in diabetic populations. This synthesis aims to consolidate current evidence, identify research gaps, and highlight the translational potential of fermented teas in diabetic cognitive impairment management.

## 2 Bioactive Compounds in Fermented Tea: Classification and Biological Functions

### 2.1 Classification of Fermented Tea

Fermented tea represents a significant category within global tea production systems. It is characterized by distinctive flavor profiles and aromatic properties that appeal to consumers, alongside documented health benefits that have garnered substantial research interest (Gu et al. 2017; Akinrinmade et al. 2017). The fermentation process involves diverse microbial communities, primarily comprising *Aspergillus niger*, *Penicillium* spp., various yeast species, and others (Tang et al. 2019; Gaggìa et al. 2018). Based on fermentation degree, teas are systematically classified into four categories: micro‐fermented, semi‐fermented, fully fermented, and post‐fermented tea (Jakubczyk et al. 2023). This established microbial basis and classification framework provide essential context for investigating fermented tea's potential bioactive modulation of the gut‐brain axis in diabetic cognitive dysfunction.

Micro‐fermented tea includes yellow tea (Hayat et al. 2015) and white tea, characterized by minimal oxidation. This classification signifies that white tea experiences minimal fermentation. Limited fermentation preserves native polyphenolic compounds, resulting in delicate sensory profiles. Scientific evidence indicates these teas exhibit antioxidant, anti‐inflammatory, and antibacterial properties (Li et al. 2022; Luo et al. 2020). The preserved native polyphenols and associated bioactive properties in micro‐fermented teas position them as key candidates for investigating gut microbiota modulation and its downstream effects on the gut‐brain axis in diabetic contexts.

Oolong tea represents the typical semi‐fermented variant (Tung et al. 2022). Partial oxidation converts polyphenols into thearubigins, yielding sensory characteristics intermediate between green and black teas. Documented health effects include neurostimulation, lipid metabolism modulation, and antioxidant activity (Tung et al. 2022; Chai et al. 2020). These distinctive biochemical transformations and multifaceted bioactivities position oolong tea as a compelling subject for investigating gut‐mediated polyphenol metabolites and their potential neuromodulatory effects within the diabetic gut‐brain axis.

Fully fermented tea, characterized by an extensive oxidation level of 80%–90%, encompasses brick tea and black tea (Liu et al. 2023; Lei et al. 2024). Polyphenol oxidation generates theaflavins and thearubigins, responsible for the characteristic reddish infusion color and robust flavor. Bioactive compounds in these teas demonstrate antioxidant capacity, antihypertensive effects, and cholesterol‐lowering potential (Pan et al. 2023; Gao, Wang, et al. 2023; Takemoto and Takemoto 2018). The high oxidation‐derived theaflavins and thearubigins in fully fermented teas represent critical bioactive candidates for exploring microbial metabolite‐mediated signaling pathways along the gut‐brain axis in diabetic cognitive impairment.

Post‐fermented tea is a distinct processing category that involves microbial solid‐state fermentation (“wet piling”) of processed green tea leaves under controlled humidity and temperature (Gao, Fu, et al. 2023). Representative varieties include Anhua black tea, Hubei Qingzhuan tea, Sichuan Tibetan tea, Guangxi Liubao tea, and Yunnan Pu'er tea (Tang et al. 2019; Assumpção et al. 2022). Additionally, fermented tea‐based beverages such as kombucha, Japanese kocha‐kin, Korean *insam‐cha* (ginseng tea), and Russian *ivan‐chai* exhibit unique fermentation dynamics and health benefits attributed to microbial transformations (Xu et al. 2019; Cheng et al. 2022; Júnior et al. 2022; Unban et al. 2019). The complex microbial consortia and biotransformation processes inherent to post‐fermented teas and traditional beverages offer a unique reservoir for investigating gut microbiota modulation and microbial metabolite‐mediated communication along the gut‐brain axis in diabetic pathology.

### 2.2 Bioactive Components in Fermented Tea: Structural Diversity and Sources

Fermentation of tea not only modifies its flavor and color but also generates diverse bioactive compounds with significant health‐promoting properties. The primary bioactive constituents of fermented tea include polyphenols, amino acids, caffeine, vitamins, minerals, and microbial metabolites (Jakubczyk et al. 2023; Banerjee and Chatterjee 2014; Gao, Wu, et al. 2022; Hu et al. 2021). These components act synergistically to confer unique health benefits, including antioxidant properties, anti‐inflammatory effects, as well as modulation of blood glucose and lipid profiles, among others (Jeong et al. 2021; Huang et al. 2019; Assad et al. 2023). This biochemical synergy, particularly microbial metabolite‐driven bioactivity, establishes fermented tea as a compelling modulator of gut‐brain crosstalk, warranting mechanistic investigation into its therapeutic potential for diabetic cognitive dysfunction.

Polyphenolic Compounds: Polyphenols represent a major class of bioactive compounds in fermented tea, predominantly comprising catechins, theaflavins, and thearubins (Musial et al. 2020; Rothenberg et al. 2018; Tong et al. 2019; Chen et al. 2019; Chen and Yang 2020). While catechins are abundant in unfermented green tea, enzymatic oxidation during fermentation converts a proportion of catechins into theaflavins and thearubins. This transformation alters the organoleptic properties of tea (e.g., flavor and color) and enhances its antioxidant capacity (Mostafa et al. 2022; Liu, Dai, et al. 2020; Sergi 2022). Theaflavins and thearubins exhibit superior free radical scavenging activity, mitigating oxidative stress and protecting cellular integrity (Liu et al. 2023; Wang et al. 2023; Xu et al. 2024). The fermentation‐enhanced antioxidant potency of theaflavins and thearubigins positions these polyphenolic metabolites as key mediators for targeting oxidative stress‐induced neuroinflammation in diabetic cognitive impairment via the gut‐brain axis.

Amino acids: Fermented tea contains elevated levels of amino acids, notably theanine (Türközü and Şanlier 2017). Theanine, a tea‐specific amino acid, demonstrates neuroactive properties, including stress reduction and sleep quality improvement (Nobre et al. 2008; Zhu et al. 2016). Although fermentation may reduce theanine content, its bioactivity toward the central nervous system is retained.

Caffeine: Fermentation reduces caffeine concentration in tea; however, residual caffeine retains mild stimulatory effects that enhance alertness, focus, and cognitive function (Fortunato et al. 2024; Gramza‐Michałowska 2014). While moderate caffeine intake is generally safe, caffeine‐sensitive individuals should exercise caution (Hayat et al. 2015). This nuanced caffeine profile, modulated by fermentation yet retaining cognitive benefits, positions it as a key variable in studying gut‐brain axis communication, particularly regarding microbiota‐mediated caffeine metabolism and its impact on diabetic neurocognition.

Vitamins and Minerals: Fermented tea provides essential vitamins (e.g., vitamin C, B‐complex vitamins) and minerals (e.g., potassium, magnesium), which support physiological functions, immune regulation, metabolic processes, and electrolyte homeostasis (Tang et al. 2019). These nutrients play a crucial role in supporting the normal physiological functions of the body, enhancing the immune response, facilitating metabolic processes, and ensuring proper electrolyte balance.

Other Bioactives: Additional beneficial compounds in fermented tea include flavonoids and polysaccharides, which exhibit anti‐inflammatory, antiviral, and glucolipid metabolism‐regulating activities (Jakubczyk et al. 2023; Hu et al. 2021; Xu et al. 2020; Yang et al. 2017; Ziemlewska et al. 2024). Table 1 summarizes the functional properties and health effects associated with each bioactive component.

**TABLE 1:** Bioactive constituents in major types of fermented tea and their proposed mechanisms alleviating diabetic cognitive dysfunction.

Fermentation category	Key bioactive ingredients	Potential mechanisms relevant to diabetic cognitive dysfunction
Lightly fermented tea (e.g., white tea)	Catechins, polyphenols	Antioxidant and anti‐inflammatory activities; improving insulin sensitivity; reducing neuroinflammation and oxidative stress in brain regions involved in cognition.
Semi‐fermented tea (e.g., oolong tea)	Theaflavins, thearubins	Lipid metabolism regulation; hypoglycemic effects; neuroprotection via modulation of synaptic plasticity and reduction in amyloid‐beta toxicity.
Fully fermented tea (e.g., black tea)	Theafuscin, caffeine	Enhancement of metabolic health; cholesterol lowering; possible adenosine receptor modulation leading to improved neuronal energy metabolism and cognitive function.
Post‐fermented tea (e.g., Pu'er tea)	Microbial metabolites (e.g., gallic acid, glutamic acid)	Gut microbiota modulation; strengthening intestinal barrier integrity; subsequent reduction in systemic inflammation and endotoxemia; increased production of neuroactive metabolites (e.g., GABA, serotonin).

## 3 The Gut‐Brain Axis in Diabetic Cognitive Dysfunction: Mechanisms and Pathways

### 3.1 Gut Microbiota Composition and Bidirectional Communication With the Gut‐Brain Axis

The interplay between gut microbiota and the gut‐brain axis is critical for understanding diabetes‐associated cognitive impairments (Loh et al. 2024). Current evidence indicates that gut microorganisms profoundly modulate the host nervous system by regulating gastrointestinal function, immune responses, and endocrine signaling (Socała et al. 2021). Notably, microbially derived SCFAs can cross the blood–brain barrier and influence cognitive processes (Dalile et al. 2019). In diabetic individuals, gut dysbiosis often impairs SCFA production, potentially exacerbating cognitive decline via gut‐brain axis dysregulation (Liu, de Bruijn, et al. 2020). Additionally, gut microbiota synthesize neuroactive compounds (e.g., dopamine, serotonin), which regulate emotion and cognition through gut‐brain communication (Ojo et al. 2020). Fermented tea—rich in polyphenols and other bioactive compounds—related cognitive decline by modulating gut microbiota composition and metabolism. Investigating interactions among fermented tea, gut microbiota, and the gut‐brain axis could yield novel therapeutic strategies for diabetic cognitive impairment and deepen understanding of gut‐brain axis dynamics (Xia et al. 2019; Zhang, Zhang, et al. 2021). Thus, fermented tea represents a promising nutraceutical modulator of the microbiota‐gut‐brain axis, warranting targeted investigation into its capacity to restore microbial SCFA production, neuroactive metabolite synthesis, and gut‐brain signaling integrity in diabetic cognitive pathology.

### 3.2 Molecular Signaling Pathways of Gut‐Brain Axis Regulation

The gut‐brain axis constitutes a bidirectional neuroendocrine network linking the gastrointestinal tract and central nervous system (Socała et al. 2021). Its regulatory significance in diabetic cognitive impairment pathogenesis and management is increasingly recognized (Loh et al. 2024). Diabetes‐related cognitive decline involves multifactorial mechanisms, with gut‐brain axis disruption identified as a key contributor (MahmoudianDehkordi et al. 2018). Alterations in microbial composition may affect cognition through diverse metabolic pathways.

Gut‐brain axis signaling operates via neural, immune, and endocrine routes (Zhang et al. 2022; Momen et al. 2024). Key metabolites including SCFAs function as vital signaling molecules that enhance intestinal barrier integrity, attenuate inflammation, and improve insulin sensitivity. Moreover, neuromodulators including brain‐derived neurotrophic factor and serotonin further contribute to diabetes‐associated cognitive deficits (Rao 2013; Zhu et al. 2024). These multifaceted signaling pathways present interconnected therapeutic targets for addressing gut‐brain axis dysregulation in diabetic cognitive pathology, with fermented tea‐derived bioactivities offering potential modulation of SCFA production and neuromodulator expression.

Additionally, polyphenols and prebiotics in fermented tea help maintain gut microbial equilibrium and mucosal barrier function, suggesting therapeutic potential against diabetic cognitive decline (Khan and Mukhtar 2018; Chen et al. 2019; Chen and Yang 2020; Zhou et al. 2019). Advanced techniques (e.g., high‐throughput sequencing, metabolomics) are now mapping gut microbiome lineages associated with diabetic cognitive impairment (Xiang et al. 2021; Sun et al. 2023; Gao, Ye, et al. 2022) and elucidating host‐microbe communication pathways. Future studies should clarify molecular mechanisms by which fermented tea modulates the gut‐brain axis, thereby informing innovative strategies to prevent or treat diabetic cognitive dysfunction.

### 3.3 Pathogenic Mechanisms Linking Gut‐Brain Axis Dysfunction to Diabetic Cognitive Impairment

The gut‐brain axis mediates bidirectional communication between the central nervous system and gut microbiota, significantly influencing diabetic cognitive dysfunction progression. Current evidence suggests diabetic gut microbiome alterations may perturb central nervous system function via the gut‐brain axis (Loh et al. 2024; Longo et al. 2023), involving multiple biomolecules and pathways. For instance, microbially produced SCFAs cross the blood–brain barrier to modulate brain activity (Dalile et al. 2019). Diabetes‐related metabolic disturbances (e.g., insulin resistance) may further impair cognition by dysregulating these metabolites (Biessels and Despa 2018; Liu, Dai, et al. 2020; Zhou et al. 2022). This pathophysiological cascade—linking gut dysbiosis, impaired SCFA signaling, and metabolic dysfunction—establishes the gut‐brain axis as a critical therapeutic target, with fermented tea offering potential to concurrently modulate microbial ecology and neuroactive metabolite bioavailability in diabetic cognitive impairment.

Elevated levels of inflammatory markers (e.g., tumor necrosis factor‐α and interleukin‐6) in diabetes can translocate to the brain via circulation, inducing neuroinflammation that disrupts neuronal plasticity and survival, ultimately compromising cognition (Zhang et al. 2023; Lei et al. 2024). Bioactive compounds in fermented tea (e.g., polyphenols, caffeine, amino acids) modulate gut microbiota, suppress inflammation, and preserve neuronal integrity (Jeong et al. 2020; Tang et al. 2019; Pan et al. 2023; Unban et al. 2019), suggesting novel therapeutic avenues for diabetic cognitive decline through the gut‐brain axis (Table 2).

**TABLE 2:** Effects of representative fermented teas on gut microbiota and their potential benefits for cognitive function in diabetic conditions.

Type of fermented tea	Effects on gut microbiota	Proposed benefits for cognitive function
Pu'er tea	Increases abundance of *Bifidobacterium* spp.; enhances microbial diversity	Strengthens intestinal barrier function; reduces systemic inflammation; may improve cognitive health via gut‐brain axis modulation
Black tea	Modulates the Firmicutes/Bacteroidetes ratio; promotes SCFA‐producing bacteria	Improves glycemic control and insulin sensitivity; may attenuate neuroinflammation and support cognitive function
Blackbrick tea	Enriches *Lactobacillus* spp.; enhances beneficial microbial metabolites	Exerts anti‐inflammatory and antioxidant effects; potentially neuroprotective through reduced oxidative stress
Kombucha tea	Promotes microbial diversity; modulates composition of symbiotic bacteria	Supports gut health and intestinal homeostasis; may positively influence mood and cognition through microbial‐gut‐brain communication
Oolong Tea	Modulates gut flora balance; increases beneficial taxa	Antioxidant and anti‐inflammatory properties; may contribute to neuroprotection and metabolic health

*Note:* The proposed mechanisms are primarily derived from preclinical studies; further clinical research is needed to establish causal relationships in humans.

Abbreviation: SCFA, short‐chain fatty acid.

Future studies should employ advanced sequencing technologies to characterize gut microbiome functionality. Integrated multi‐omics approaches—including transcriptomics, metabolomics, and neuroimaging analyses—will advance understanding of gut‐brain axis influences on cognition. Identifying specific biomarkers may further guide clinical interventions.

## 4 Therapeutic Modulation of the Gut‐Brain Axis for Diabetic Cognitive Dysfunction

### 4.1 Targeting Gut Microbiota Composition

Research on the gut‐brain axis increasingly impacts fermented tea components in the regulation of gut microbiota. The diversity and balance of the gut microbiome are critical determinants of human health, significantly influencing metabolic and immune functions. Fermented tea contains diverse bioactive compounds, including polyphenols, polysaccharides, amino acids, and alkaloids, which contribute to shaping the intestinal environment and modulating microbial communities. Particularly, catechins and other phenolic constituents in fermented tea promote the proliferation of probiotics while suppressing pathogenic strains, thereby fostering a more favorable gut microbiome profile (Khan and Mukhtar 2018; Tang et al. 2019; Júnior et al. 2022). Furthermore, metabolites derived from fermented tea, including SCFAs, modulate microbial composition and function, enhance intestinal barrier integrity, and mitigate intestinal inflammation through pH regulation (Song et al. 2022; Fan et al. 2013). These molecular interactions extend systemically, potentially influencing cognitive function via circulatory or neurotransmission pathways connecting the gut to the brain (Li, Zhang, et al. 2023). Alterations in gut microecology have been correlated with cognitive impairment in diabetes, as dysbiosis in diabetic patients may disrupt neuroinflammatory pathways through metabolite dysregulation, exacerbating cognitive decline (Lu et al. 2022; Ng et al. 2021). Consequently, interventions including fermented tea consumption may confer therapeutic benefits for diabetic cognitive impairment by indirectly or directly ameliorating gut flora composition. These findings highlight the potential of fermented tea components in mitigating diabetes‐associated cognitive dysfunction through modulation of the gut microbiota.

### 4.2 Influence of Microbial Metabolites

Evidence suggests that bioactive compounds in fermented tea critically modulate interactions with the gut microbiota (Sun et al. 2021). These compounds not only promote the growth of beneficial bacteria but also inhibit the function of specific pathogenic strains, thereby enhancing intestinal health. Certain gut microbes metabolize polyphenols to produce SCFAs, which regulate intestinal barrier integrity, reduce inflammation, and significantly contribute to gut–brain axis homeostasis (Zhang, Cheng, and Zhang 2021; Xie et al. 2022). Crucially, butyrate and its derivatives regulate G protein‐coupled receptor (GPCR) activity, influencing insulin sensitivity and glucose metabolism; this is a mechanism potentially relevant for interventions targeting diabetes‐related cognitive decline (Mayorga‐Ramos et al. 2022). Moreover, these metabolites can cross the blood–brain barrier to directly influence brain function, eliciting anti‐inflammatory responses within the central nervous system (Stilling et al. 2016). The targeted modulation of gut microbiota by fermented tea bioactives suggests its dietary incorporation represents a non‐pharmacological strategy to influence microbial composition and metabolic output, potentially ameliorating diabetes‐associated cognitive issues. Consequently, elucidating the precise relationships between fermented tea constituents and gut microbiota is essential for advancing understanding of the gut–brain axis in clinical contexts and developing innovative therapeutic approaches for diabetic complications.

### 4.3 Enhancing Intestinal Barrier Integrity

Bioactive components in fermented tea, including catechins, polysaccharides, and organic acids, interact with gut microbiota to preserve intestinal mucosal barrier integrity. These compounds promote beneficial probiotics (e.g., *Bifidobacterium*, *Lactobacillus*) while inhibiting detrimental bacteria, supporting gut ecosystem equilibrium (Gao, Wang, et al. 2023). By reinforcing the intestinal barrier, fermented tea contributes to the management of diabetic cognitive dysfunction, impacting inflammatory responses vital for gut‐brain axis communication. Studies demonstrate that fermented tea constituents upregulate tight junction protein expression, improve barrier function, and reduce circulating endotoxin and inflammatory markers, thereby conferring neuroprotection and potentially enhancing cognitive capabilities (Jakubczyk et al. 2023; Qin et al. 2010; Prasanth et al. 2019; Mhd Jalil et al. 2019). Understanding this mechanism provides insights for addressing diabetic complications and supports the application of fermented tea in functional foods and pharmaceutical development.

### 4.4 Modulation of Neural Signaling Pathways

Fermented tea, recognized for its nutritional value, has garnered significant scientific interest for its potential in addressing diabetic cognitive dysfunction. Key active constituents, such as catechins and polyphenols, exert effects on neural signaling pathways (Sun et al. 2021; Uniyal et al. 2021). These pathways enhance cerebral insulin signaling, support neuronal function and plasticity, activate the sympathetic nervous system, and modulate gut microbiota composition, collectively improving nervous system function via the gut‐brain axis (Xu et al. 2021; Zhang, Cheng, and Zhang 2021). Fermented tea components may promote myelination, protect neurons from oxidative stress, and alleviate diabetic neuropathy through anti‐inflammatory mechanisms (Ojo et al. 2020; Sabu et al. 2002). Furthermore, polyphenols and prebiotics regulate the gut milieu, fostering beneficial bacteria growth and indirectly influencing cognition by modulating gut‐derived signaling molecules (e.g., SCFAs). Elucidating these processes clarifies the benefits of fermented tea and reveals novel therapeutic avenues for mitigating cognitive decline in diabetic patients.

### 4.5 Immunomodulation and Control of Inflammation

Bioactive compounds in fermented tea (e.g., polyphenols, alkaloids) may ameliorate diabetic cognitive dysfunction via gut‐brain axis modulation. These components effectively regulate gut microbiota composition (Van Buiten et al. 2018; Teixeira Oliveira et al. 2023), beneficially impacting intestinal barrier integrity. Enhanced barrier function reduces systemic inflammatory agent dissemination and dampens inflammatory signaling to the brain (Ng et al. 2021; Lee et al. 2023). Antioxidant tea polyphenols synergize with microbially derived SCFAs to modulate immune responses, crucially mitigating gut oxidative stress, a key factor in diabetes‐associated neuroinflammation (Kim et al. 2020, 2021). Fermented tea also supports glycemic control, reduces insulin resistance, and enhances insulin sensitivity, contributing to cognitive function recovery (Lee et al. 2015). These findings underscore fermented tea's significant role in regulating the gut‐brain axis, modulating inflammation, and influencing immune responses in diabetic cognitive impairment, suggesting promising therapeutic strategies for future investigation.

### 4.6 Regulation of Endocrine and Metabolic Pathways

**FIGURE 1:**
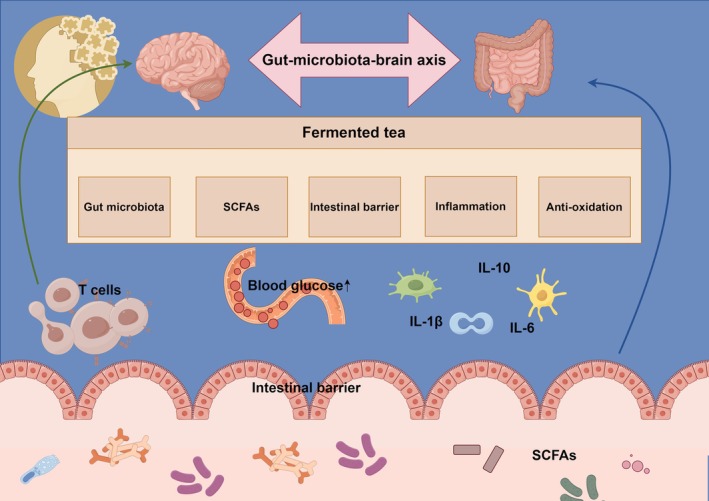
Proposed mechanistic schema illustrating how fermented tea mitigates diabetes‐associated cognitive dysfunction via the gut–brain axis. Chronic hyperglycaemia disrupts intestinal homeostasis, leading to dysbiosis (↓ beneficial taxa, ↑ pathobionts), decreased microbial production of short‐chain fatty acids (SCFAs; acetate, propionate, butyrate), and compromised intestinal barrier integrity (↑ permeability, ↓ tight‐junction proteins). Subsequent translocation of lipopolysaccharide (LPS) triggers systemic low‐grade inflammation (↑ IL‐1β, IL‐6, TNF‐α) and oxidative stress (↑ ROS), which propagate neuroinflammation and impair synaptic plasticity, ultimately manifesting as cognitive decline. Fermented tea—rich in catechins, theaflavins, thearubigins, and microbial metabolites—restores eubiosis (↑ Bifidobacterium, *Lactobacillus, Blautia*), enhances SCFA biosynthesis, and reinforces the intestinal barrier (↑ ZO‐1, occludin). These effects attenuate endotoxaemia, suppress peripheral and neuro‐inflammation (↑ IL‐10), and reduce oxidative injury. Concurrently, tea polyphenols and their microbial catabolites cross the blood–brain barrier (BBB) to activate cerebral insulin signaling (↑ PI3K/Akt), up‐regulate brain‐derived neurotrophic factor (BDNF) and serotonin pathways, thereby preserving neuronal integrity and improving cognitive performance in diabetic individuals.

Fermented tea has attracted considerable attention for its potential to mitigate diabetes‐associated cognitive impairment. Analysis of its components and gut‐brain communication pathways reveals that bioactive compounds regulate endocrine function, thereby influencing nervous system activity (Yang et al. 2017; Ma et al. 2022), with positive implications for cognition. For instance, polyphenols enhance insulin sensitivity in diabetic patients, improving metabolic health and cognitive performance (Li et al. 2022; Sabu et al. 2002; Miyata et al. 2013). Moreover, microbially produced SCFAs (derived from amino acid and polysaccharide fermentation) cross the blood–brain barrier to influence the central nervous system, regulating neural pathways involved in learning and memory. Fermented tea also contains bioactives affecting neurotransmitter production and release, significantly contributing to synaptic transmission and cognitive maintenance (Pavlović et al. 2023), as shown as Figure 1. Critically, the efficacy of fermented tea likely arises from the synergistic action of multiple bioactive compounds, not a single constituent (Tang et al. 2019). Therefore, further research is required to establish dose–response relationships and identify optimal bioactive combinations for clinical applications. Developing novel interventions for diabetic cognitive decline necessitates thorough investigation of fermented tea component interactions along the gut‐brain axis to facilitate improved therapeutic strategies.

## 5 *Chibi Green Brick Tea*: Potential Therapeutic Agent for Diabetic Cognitive Dysfunction via the Gut‐Brain Axis

*Chibi Green Brick tea*, a historically significant fermented tea originating from Chibi City, Hubei Province, China, is distinguished by its unique production process involving pile fermentation, aging, and artisanal refinement (Xu et al. 2019; Yang et al. 2017). Renowned for its bluish‐brown appearance, complex aroma, rich flavor, and vibrant liquor, *Chibi Green Brick tea* holds cultural prominence in Chinese tea traditions. Recent research highlights its therapeutic promise in diabetes management and associated complications, notably diabetic cognitive dysfunction.

*Chibi Green Brick tea* aqueous extract enhances systemic antioxidant capacity and mitigates metabolic syndrome via activation of the Nrf2 signaling pathway (Cheng et al. 2022). This mechanism reduces oxidative stress and may protect against diabetes‐associated neurodegeneration, suggesting relevance to diabetic cognitive decline pathogenesis. Furthermore, the extract from *Chibi Green Brick tea* demonstrates efficacy in glycemic control, attenuation of hepatic oxidative stress, and amelioration of insulin resistance (Cheng et al. 2022; Gao et al. 2018). These effects may beneficially modulate metabolic pathways implicated in cognitive decline.

Emerging evidence underscores the broad bioactivity of *Chibi Green Brick tea*, including hypolipidemic, anti‐obesity, hypoglycemic, uric acid‐lowering, hepatoprotective, gastrointestinal regulatory, anti‐radiation, and anti‐aging properties (Li, Yin, et al. 2023). Technological advances in fermentation and aging now enable the accelerated production of high‐quality *Chibi Green Brick tea* comparable to traditionally aged variants.

*Chibi Green Brick tea* modulates gut microbiota composition by enriching beneficial taxa (*Bacteroides*, *Blautia*, *Clostridium*) while suppressing pathobionts (*Coliforms*, *Faecalis*, *Lactobacillus*) (Wei et al. 2023). This regulation promotes the synthesis of short‐chain fatty acids (e.g., acetate, propionate), which support intestinal barrier integrity, epithelial energy metabolism, and gut‐brain axis communication, a critical pathway in neurocognitive health.

**FIGURE 2:**
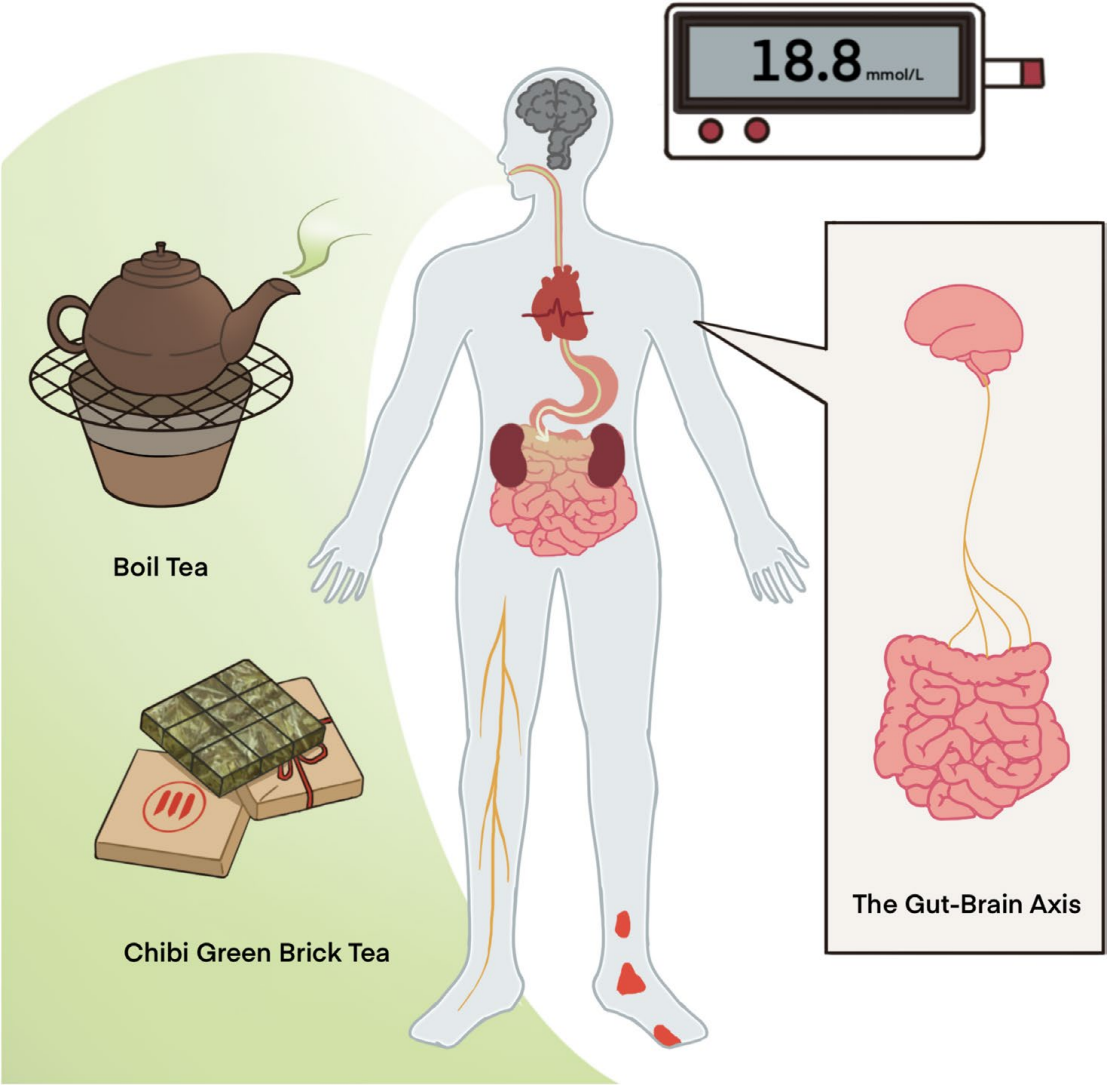
*Chibi Green Brick tea* orchestrates a multi‐targeted gut–brain axis intervention against diabetic cognitive dysfunction. *Chibi Green Brick tea* aqueous extract exerts prebiotic‐like activity by selectively enriching SCFA‐producing commensals (*Bacteroides*, *Clostridium* cluster XIVa) and suppressing pro‐inflammatory *Enterobacteriaceae*, which restores microbial‐derived SCFA levels. Elevated butyrate and propionate enhance colonic epithelial energy metabolism (↑ GPR41/43 signaling), strengthen tight‐junction complexes, and reduce circulating LPS and inflammatory cytokines (↑ IL‐10, ↓ IL‐6, TNF‐α). Downstream, diminished systemic inflammation mitigates microglial activation and hippocampal oxidative stress (↑ Nrf2, ↓ NF‐κB). Metabolically, *Chibi Green Brick tea* polyphenols improve peripheral insulin sensitivity (↑ GLUT4 translocation) and stabilize blood glucose, indirectly alleviating glucotoxic neuronal injury. Collectively, these *Chibi Green Brick tea*‐induced microbiota–gut–brain interactions preserve synaptic plasticity and cognitive function in diabetic models.

Although direct evidence of the effects of *Chibi Green Brick tea* on diabetic cognitive dysfunction remains limited, its established benefits in metabolic regulation, antioxidant defense, and gut microbiota modulation suggest potential neuroprotective actions via the gut‐brain axis (Figure 2). Future research is needed to explore the direct effects of *Chibi Green Brick tea* on diabetic cognitive dysfunction and elucidate the underlying mechanisms through the gut‐brain axis. It is also essential to advance detection methods to ensure the quality and safety of *Chibi Green Brick tea* (Wei et al. 2023).

As a natural adjunctive therapy, *Chibi Green Brick tea* holds promise for diabetes and diabetic cognitive dysfunction management. Research should prioritize identifying active constituents responsible for cognitive enhancement and validating clinical efficacy through rigorous trials.

## 6 Discussion

DM significantly accelerates the progression from mild cognitive impairment to Alzheimer's disease (AD), particularly within the first year after mild cognitive impairment diagnosis. DM exacerbates atrophy in key brain regions such as the nucleus accumbens, reduces gray matter volume, and decreases sulcal depth, correlating with sharper cognitive decline. These findings highlight shared pathophysiological mechanisms, like insulin resistance and neuroinflammation, and emphasize the need for early intervention during this critical window to delay AD progression in diabetic patients. A recent systematic review comprehensively examines the role of the gut microbiome through the microbiota‐gut‐brain axis, highlighting mechanisms such as neuroinflammation, metabolic dysregulation, and immune modulation. It emphasizes therapeutic strategies including probiotics, prebiotics, synbiotics, fecal microbiota transplantation, and traditional Chinese medicine, which collectively aim to restore microbial balance and mitigate AD pathology. Although fermented tea is not explicitly discussed in the review, its high content of bioactive compounds, including polyphenols, catechins, and microbial metabolites, aligns with the described mechanisms. Fermented tea may modulate gut microbiota composition, enhance short‐chain fatty acid production, strengthen intestinal barrier function, and reduce systemic inflammation and oxidative stress, thereby potentially protecting against cognitive decline.

This comprehensive review examines fermented teas and their potential to mitigate diabetes‐associated cognitive impairment, with a specific focus on the gut‐brain axis. The study underscores the promising therapeutic role of fermented teas in modulating gut microbiota and enhancing cognitive well‐being. Diverse fermented tea varieties, their bioactive constituents, and mechanisms influencing cognitive function in diabetic populations are systematically evaluated.

Culturally embedded products, exemplified by *Chibi Green Brick tea*, possess not only historical significance but also demonstrate bioactive properties with therapeutic potential. Their efficacy in ameliorating diabetes, hyperlipidemia, and obesity highlights their multifaceted health benefits. As a functional beverage, fermented tea offers a practical and safe strategy for combating cognitive decline in diabetic patients, owing to its simple preparation, ease of consumption, and favorable safety profile. While the therapeutic promise of fermented tea is considerable, several limitations warrant acknowledgment. Methodological variations in the preparation and consumption practices across cultures introduce heterogeneity in dosage and bioefficacy. Furthermore, the specific microbial consortia and optimal fermentation parameters required to maximize health benefits remain to be fully characterized. This knowledge gap may impact the consistency and reliability of fermented tea as a therapeutic intervention for diabetes‐related cognitive dysfunction.

The review emphasizes the significance of fermented tea as a natural, microbiota‐targeting approach to enhance cognitive function in diabetic individuals via the gut‐brain axis. It highlights the critical need for further research to harness these therapeutic benefits and translate findings into clinical practice. Future investigations should prioritize: standardizing fermentation protocols; conducting controlled clinical trials to establish evidence‐based dosages; and elucidating the precise molecular and microbial mechanisms underpinning the effects of fermented tea on cognitive health. The potential of fermented tea as a natural, accessible intervention for diabetes‐associated cognitive impairment merits sustained scientific scrutiny and dedicated research investment.

## 7 Conclusions

Fermented tea, rich in bioactive compounds, represents a promising therapeutic avenue for mitigating diabetes‐associated cognitive impairment. This potential is mediated through modulation of the gut‐brain axis and subsequent influences on gut microbiota. The distinct properties of *Chibi Green Brick tea* warrant comprehensive investigation to elucidate its mechanisms of action and optimize its clinical translation. Future research should prioritize establishing standardized fermentation protocols and implementing rigorously controlled clinical trials to validate the efficacy and safety of fermented teas in addressing the intricate interplay between diabetes, cognitive decline, and gut microbiome dysregulation.

## Author Contributions

**Ruyi Zhang:** writing – original draft (equal). **Wenli Liao:** writing – review and editing (equal).

## Consent

All authors agree to the publication of this study.

## Conflicts of Interest

The authors declare no conflicts of interest.

## Data Availability

No data was used for the research described in the article.
